# Marshall Properties and Thermal Conductivity of Asphalt Mixtures Modified with Polyurea-Shelled n-Nonadecane Phase-Change Nanocapsules

**DOI:** 10.3390/polym18212635

**Published:** 2026-10-24

**Authors:** Semahat Doruk, Başak Varlı Bingöl, Mehmet Emin Özdemir, Murat Yaylacı

**Affiliations:** 1 Department of Chemical Engineering, Çankırı Karatekin University, 18100 Çankırı, Türkiye; sbarlak@karatekin.edu.tr; 2 Department of Civil Engineering, Çankırı Karatekin University, 18100 Çankırı, Türkiye; basakvarlibingol@karatekin.edu.tr (B.V.B.); meozdemir@karatekin.edu.tr (M.E.Ö.); 3 Department of Civil Engineering, Recep Tayyip Erdogan University, 53100 Rize, Türkiye; 4 Dijitalpark Teknokent, Murat Yaylacı-Luzeri R&D Engineering Company, 53100 Rize, Türkiye; 5 Turgut Kıran Maritime Faculty, Recep Tayyip Erdogan University, 53900 Rize, Türkiye

**Keywords:** flexible pavement, phase change material, nanocapsule, n-Nonadecane, thermal conductivity, Marshall mix design, analysis of variance

## Abstract

The rise in the surface temperatures of flexible pavements, driven by climate change and urbanization, increases the need for materials with a modified thermal response while retaining acceptable mixture properties. In this study, a previously developed n-nonadecane capsule system with a polyurea (PUA) shell was incorporated into bitumen at 1, 3, and 5% by weight of the binder, and the resulting asphalt mixtures were evaluated at the capsule/binder and mixture scales. The capsule system was characterized by SEM and FTIR, and its thermal behavior by DSC and TGA; the effective thermal conductivity of compacted mixtures was measured between 20 and 40 °C with a KD2 Pro thermal analyzer using a KS-1 transient needle probe, and Marshall volumetric and mechanical responses were determined on three replicate specimens per group. DSC showed melting at 34.5 °C for pure n-nonadecane and at ~33.8 °C for the capsule, with total endothermic enthalpies over the 20–40 °C interval of 212.2 and 92.8 J/g, corresponding to an enthalpy-based encapsulation ratio of ~44%. TGA showed no appreciable thermal decomposition up to the asphalt mixing temperature, but this measurement does not establish mechanical capsule survival during mixing and compaction. The measured effective thermal conductivity decreased with both temperature and capsule content, from 1.76 W/m·K for the neat mixture to 1.31 W/m·K for PCM-5 at 40 °C. Marshall stability was higher than the control at all tested dosages and reached 14.01 kN at PCM-3 versus 10.42 kN for the control; however, the volumetric response also shifted with capsule addition, and the modified mixtures were not independently optimized at each dosage. Accordingly, the present results are interpreted as formulation responses under a common 6.0% binder-content condition rather than as proof of an optimized pavement design or field-scale thermal regulation.

## 1. Introduction

Flexible pavements are exposed not only to traffic loads but also to environmental temperature variations. High ambient temperatures reduce asphalt binder viscosity and cause permanent deformation through rutting, which reduces riding comfort, increases maintenance costs, and threatens traffic safety. Driven by global climate change and the urban heat island effect, rising pavement surface temperatures have increased the need for new-generation materials that improve the thermal behavior of asphalt mixtures [1,2,3,4,5].

Phase-change materials (PCMs) store latent heat by changing phase within a narrow temperature range and can therefore buffer ambient temperature fluctuations. Because latent-heat storage offers a much higher energy density than sensible-heat storage, PCMs are advantageous for thermal energy storage applications [6,7,8,9,10]. PCMs are generally classified as organic, inorganic, and eutectic; organic PCMs such as paraffins and n-alkanes are preferred because of their chemical stability and suitable phase-change temperatures, whereas inorganic PCMs such as salt hydrates may suffer from supercooling and phase segregation [6,11].

Incorporating PCMs into bitumen improves the pavement’s thermal regulation capacity by absorbing and releasing energy during temperature changes and reducing thermal stresses [2,3,12]. Reported systems include microencapsulated paraffins, composite shape-stabilized PCMs, expanded-graphite/PEG composites, PCM-impregnated ceramsite, and nanoparticle-based additives [13,14,15,16]. In addition, several studies report that adding PCM or capsules improves the viscosity, stiffness, and deformation resistance of the binder [14,17]; this places PCM alongside polymeric, fibrous, and waste-derived additives increasingly used to tailor bitumen and asphalt-mixture performance [18,19]. Field-scale studies have shown that PCM-modified pavements exhibit greater resistance to temperature fluctuations and can mitigate the urban heat island effect [1,3,16,20], while PCM addition has also been reported to reduce low-temperature cracking and freeze–thaw damage and to extend pavement service life [21].

However, direct use of PCMs is limited by leakage, low mechanical strength, chemical instability, and insufficient thermal stability. To overcome these problems, researchers encapsulate PCMs in a polymeric or inorganic shell [22,23]. Polymeric shells such as polyurethane protect the core material from external effects, prevent leakage, improve compatibility with bitumen, and provide controlled heat transfer [3,24]. Reducing capsule size from the micro- to the nanoscale may increase interfacial area and influence dispersion behavior in the host matrix; however, the rheological consequences of particle size depend on the specific binder–particle system and were not measured directly in the present study. Nanoencapsulated PCMs have been investigated in heat-transfer fluids, polymer foams, nanofluids and composite materials [7,25,26,27,28,29]. Nevertheless, most previous studies have used PCMs either unencapsulated or only at the microcapsule scale, and studies on nanoencapsulated PCM-modified asphalt remain scarce. Moreover, researchers rarely report the thermal benefits of PCM modification alongside a statistically supported evaluation of the mixture-scale volumetric and mechanical response, even though thermal improvement is only practically valuable if the mixture still meets the mix-design criteria.

The aim of this study is therefore to apply a previously developed polyurea-shelled, n-nonadecane-core PCM nanocapsule system to bitumen at 1, 3 and 5% by weight of the binder and to determine the effect of this addition on (i) capsule-scale phase-change behavior and thermal stability, (ii) the effective thermal conductivity of compacted asphalt mixtures, and (iii) their Marshall volumetric and mechanical properties. The latter were evaluated by one-way analysis of variance and Tukey’s honestly significant difference procedure to compare the tested formulations under a common binder-content condition. Although the thermal conductivity of PCM-modified asphalt has recently attracted attention [30], it has rarely been evaluated over a service-relevant temperature range together with the Marshall response of the same mixture formulations. The thermal-conductivity specimens and the Marshall specimens were separate experimental sets.

## 2. Materials and Methods

### 2.1. Materials

**Figure 1:**
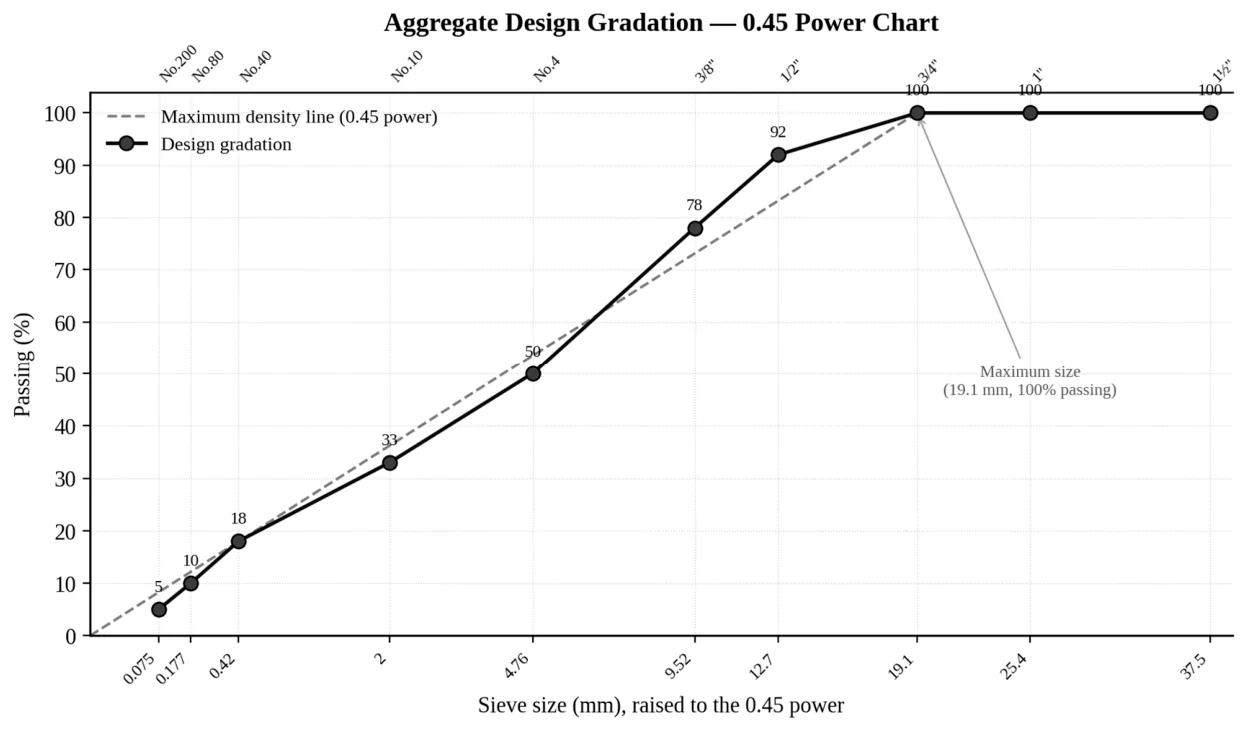
Aggregate design gradation plotted on the 0.45 power (maximum density) chart. The dashed line is the maximum density line drawn from the origin to the maximum aggregate size (19.1 mm at 100% passing); numeric labels indicate the percent passing at each sieve.

n-Nonadecane, whose phase-change temperature lies in the range relevant to pavement service conditions and which has been used previously as a nanoencapsulated core material [22,25], was used as the core material, and polyurethane as the shell material; toluene diisocyanate (TDI), diethylenetriamine (DETA), cyclohexane, and the non-ionic surfactant NP-10 were used in the synthesis. Crushed limestone was used for the coarse aggregate, fine aggregate, and mineral filler. All fractions were sourced from a single supplier at a quarry in Ankara, Turkey. A 50/70 penetration-grade bitumen obtained from an asphalt plant in Ankara, Turkey, served as the base binder. The physical properties of the bitumen and of the aggregate, including its coarse, fine, and mineral-filler fractions, used in the preparation of the Marshall specimens were determined in accordance with the ASTM standards listed in Table 1, and the aggregate blend was proportioned to the 0.45 power maximum density gradation used in the Marshall mix-design procedure [31]; the design gradation is shown in Figure 1.

### 2.2. Synthesis of the Nanocapsules

**Figure 2:**
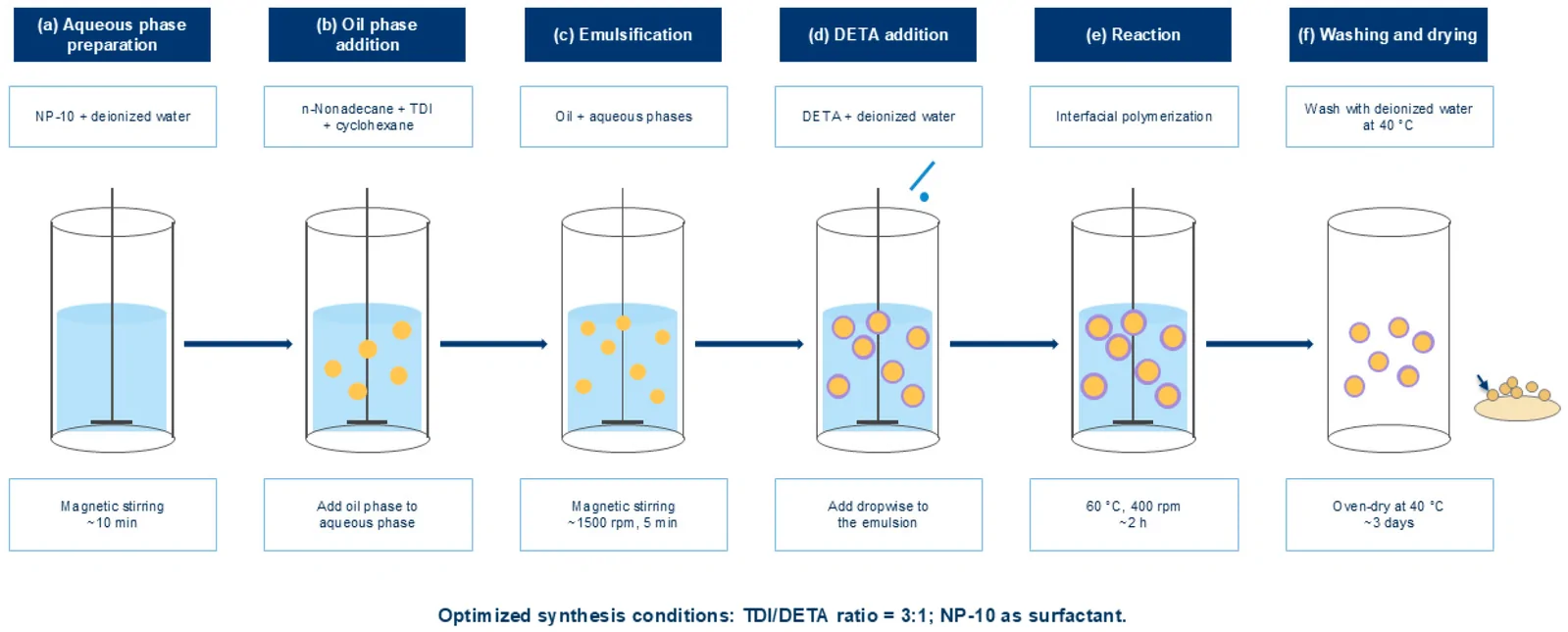
Schematic of the interfacial polymerization route used to synthesize the PU–n-nonadecane nanocapsules.

The authors synthesized the PCM nanocapsules by interfacial polymerization using the n-nonadecane capsule route previously developed, optimized, and characterized [25,26,27]. The aqueous phase was prepared by dissolving 0.5 g of the non-ionic surfactant NP-10 in 20 mL of deionized water under magnetic stirring for about 10 min. The oil phase was prepared by dissolving 4 g of n-nonadecane (core material) in 10 mL of cyclohexane, then adding 3 g of toluene-2,4-diisocyanate (TDI, shell precursor). The two phases were then combined and emulsified by magnetic stirring at approximately 1500 rpm for 5 min; no ultrasonic treatment was applied at any stage. A solution of 1 g of diethylenetriamine (DETA) in 10 mL of deionized water was added dropwise to the emulsion while the stirring rate was reduced to 600–700 rpm. The interfacial polymerization was then carried out at 60 °C for approximately 2 h. The product was filtered, washed with deionized water at 40 °C, and dried in an oven at 40 °C for approximately 3 days. The TDI/DETA mass ratio was therefore 3:1, corresponding to a molar ratio of approximately 1.8:1, and NP-10 was used as the surfactant, in accordance with conditions previously optimized for this capsule system [25,26,27]. The dried product from this batch weighed 5.8 g, corresponding to a mass yield of approximately 73% relative to the 8 g of n-nonadecane, TDI, and DETA charged. The overall synthesis route is illustrated in Figure 2.

The formulation contains no polyol, so the shell-forming step is an isocyanate–amine polyaddition rather than a urethane-forming polycondensation. Three reactions are expected at the oil/water interface: (i) the primary amine groups of DETA react with the –NCO groups of TDI to form urea linkages; (ii) isocyanate groups that reach the aqueous phase hydrolyze to amines, which react with further TDI and again yield urea; and (iii) the hydroxyl-terminated surfactant NP-10 present at the interface may contribute a limited number of urethane linkages. Urea linkages are therefore expected to dominate the shell, and the shell is designated polyurea (PUA) throughout this work. Earlier reports of this formulation described the same capsule system as polyurethane [25,26,27,29], assigning the infrared band near 1643–1650 cm^−1^ to a urethane carbonyl. Both the designation and that band assignment are revised here. The carbonyl absorption of a urethane group lies at 1700–1730 cm^−1^, whereas the observed position, taken together with the N–H stretching band near 3300 cm^−1^ and the amide II band near 1540 cm^−1^, corresponds to a urea carbonyl (amide I). This revision follows the reaction chemistry set out above and the spectroscopic evidence presented in Section 3.1, and it concerns only the identification of the shell polymer: the spectra, thermal properties, and particle-size data reported in those earlier studies are measurements of the same material and are unaffected. The urethane fraction arising from route (iii) was not quantified.

### 2.3. Preparation of the Pcm-Modified Binder

Modified binders were prepared separately for capsule/binder characterization and for Marshall specimen production. In the first stage, the synthesized nanocapsules were blended into the neat bitumen at 1, 3, and 5% by weight of the binder at about 100–110 °C using a high-shear mechanical mixer, in order to pre-disperse the capsules in the binder at a temperature close to the melting range of the core material; the samples were then cooled and used for the capsule- and binder-scale characterization. For each Marshall specimen, an independent modified binder batch was prepared. The PCM nanocapsules were added to the bitumen at approximately 100–110 °C. The blend was then heated to 150 °C without stirring, followed by mechanical mixing at 2000 rpm for approximately 2 min. No binder batch was shared between specimens. The 1, 3, and 5% capsule levels were selected to provide low, intermediate, and relatively high modification levels for comparison across a controlled dosage series; they were not intended to represent previously optimized dosages.

### 2.4. Marshall Specimen Preparation and Testing

The aggregates, batched to the design gradation shown in Figure 1, were oven-dried at 150 °C before mixing. The pre-heated aggregate was then combined with the binder (neat or PCM-modified, as appropriate) and mixed until the aggregate particles were thoroughly and uniformly coated. The binder content was fixed at 6.0% by weight of the total mixture for all four groups, this being the optimum binder content determined for the neat control mixture by the Marshall design procedure, and the PCM dosages (1, 3 and 5%) are expressed as percentages by weight of this bitumen. The PCM mixtures were therefore compared with the control at a common binder content rather than each at its own optimum. The resulting loose mixture was compacted at 150 °C into standard Marshall specimens by applying 75 blows to each face, corresponding to the compaction level specified for heavy traffic in the Marshall mix-design method [30]; Marshall stability and flow remain the most widely used indicators for comparing modified and unmodified mixtures [19,38,39]. Three replicate specimens (n = 3) were prepared for each of the four mixture groups; each replicate was produced from an independently batched mixture (the aggregate fractions and the binder were weighed out and mixed separately for every specimen), so that each specimen constituted an independent experimental unit rather than a subsample of a single loose-mix batch. After compaction and cooling, the specimens were tested at 60 °C in accordance with ASTM D6927 [40]; this test temperature is above the PCM melting point, so the PCM was in its molten (fully phase-changed) state during testing. The volumetric properties (air voids, VMA and VFA) and the Marshall stability and flow were determined. The Marshall quotient was computed individually for each specimen as the ratio of its Marshall stability to its flow (MQ_i_ = S_i_/F_i_, where the subscript i denotes the individual specimen), and the value reported for each mixture is the mean of the three individual specimen quotients rather than the ratio of the mean stability to the mean flow.

### 2.5. Characterization and Thermal-Conductivity Measurement

The morphology of the nanocapsules and their distribution within the bitumen matrix were examined by scanning electron microscopy at the Central Research Laboratory of Çankırı Karatekin University (ÇANKAM), Çankırı, Türkiye, using a Carl Zeiss Sigma 300 VP (ZEISS, Oberkochen, Germany). Fourier-transform infrared spectroscopy was performed at the same facility using a Bruker Tensor II spectrometer (Bruker, Ettlingen, Germany) in attenuated-total-reflectance mode. Four materials were measured: the toluene-2,4-diisocyanate shell precursor, the PUA-PCM nanocapsules, the pure n-nonadecane core and the neat 50/70 bitumen. The spectra were used to identify the shell polymer; they were not used to quantify capsule content in the modified binder, for the reason given in Section 3.1. The reading-level spectra are provided in the Supplementary Material. Thermal stability was evaluated by thermogravimetric analysis at ÇANKAM using a TA Instruments SDT Q600 analyzer (TA Instruments, New Castle, DE, USA). Phase-change temperatures and enthalpies were determined by differential scanning calorimetry at the Central Laboratory of Middle East Technical University (METU), Ankara, Türkiye, using a PerkinElmer Diamond DSC (Waltham, MA, USA). The SEM, FTIR, DSC and TGA measurements reported for the capsule scale were all performed on the nanocapsule batch described in Section 2.2; the DSC records for the capsule, the pure n-nonadecane core and the empty-shell reference are provided in the Supplementary Material. These capsule/binder-scale measurements characterize the material system before full aggregate mixing and Marshall compaction and are not used as direct proof of post-compaction capsule integrity. The empty-shell reference shown in the DSC comparison was used only as a capsule-scale characterization control and was not used as an asphalt-mixture control.

**Figure 3:**
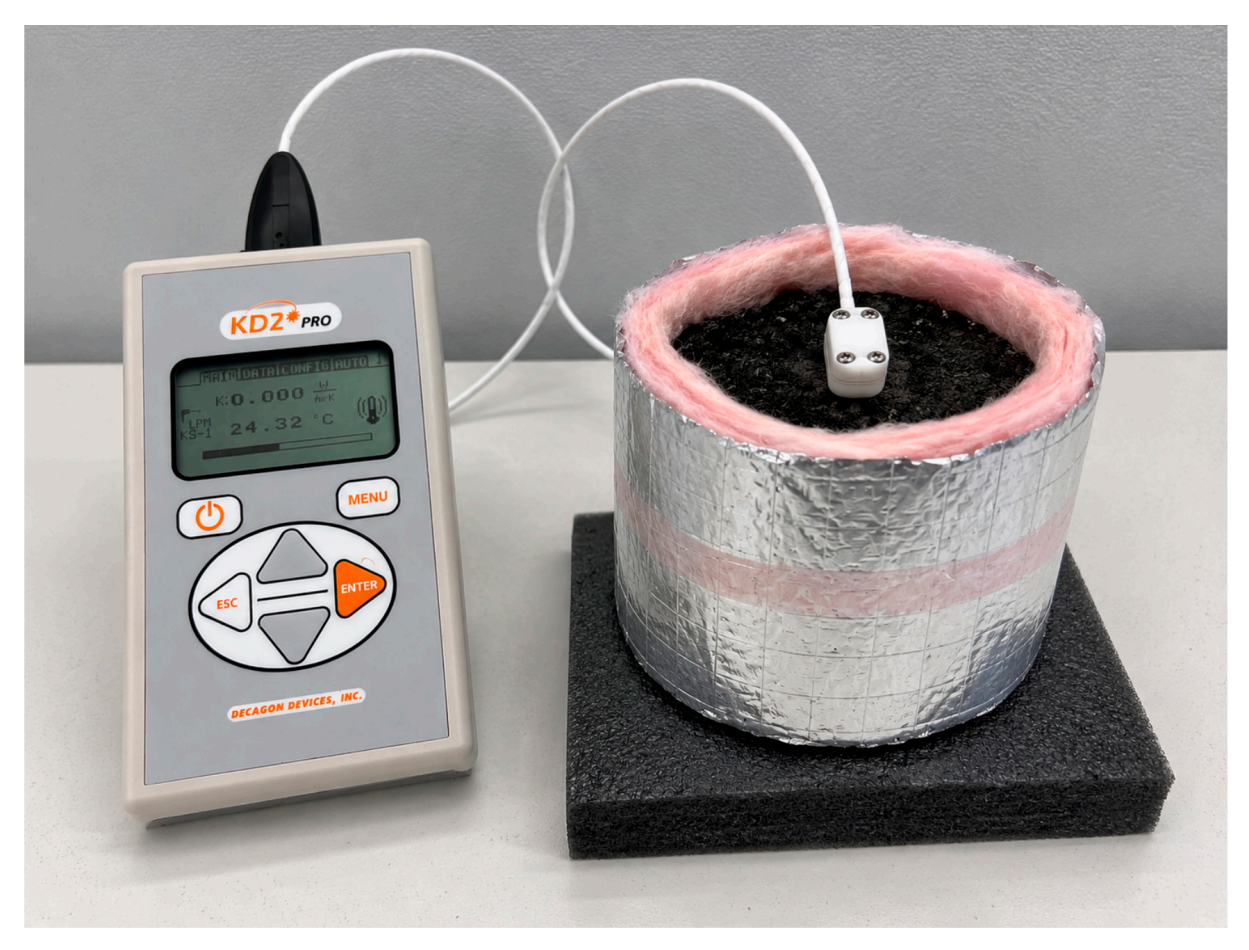
Thermal-conductivity measurement setup: KD2 Pro analyzer with the KS-1 needle sensor inserted into the thermally insulated specimen.

The effective thermal conductivity (λ) of the mixtures was measured between 20 and 40 °C on separate compacted specimens produced using the same mixture formulations and batching, mixing and compaction procedure as the Marshall specimens (Section 2.4). For each mixture–temperature condition, a single specimen was prepared and compacted, for a total of twenty specimens (four mixtures × five temperatures); each specimen was measured at one temperature only. Immediately after compaction, while the specimens were still warm, needles having the same diameter as the KS-1 probe were inserted to form probe channels. After cooling, these needles were removed, and the preformed channels were used for KS-1 insertion; no thermal contact paste or gel was used. Before measurement, the specimens were conditioned in an oven to the intended test temperature, removed, and wrapped in thermal-insulation material to minimize heat loss. The KD2 Pro (Decagon Devices, Pullman, WA, USA) reading was used to verify the actual measurement temperature (Figure 3). If the indicated temperature was above the intended value, the specimen was allowed to cool before testing; if it was below the intended value, it was reheated. Four consecutive readings were collected from the same preformed probe channel in each specimen and were treated as technical replicates. The KD2 Pro recorded the temperature with each reading; the recorded values remained within ±0.7 °C of the nominal level (for example, 20.1–20.7 °C at the 20 °C level). No second specimen was prepared for any mixture–temperature combination, so the protocol provides technical repetition but no between-specimen replication. Measurements were made with a KD2 Pro thermal analyzer (Decagon Devices, Inc., Pullman, WA, USA.) equipped with a KS-1 single-needle sensor applying the transient line-heat-source principle described in ASTM D5334 [41]. Transient needle-probe methods of this type are widely used for asphalt materials because they require no steady state and impose only a small temperature rise on the specimen [42,43]. Higher-temperature exploratory measurements were attempted above 40 °C, but as the asphalt specimens softened, the measurements became increasingly unstable, with increased instrument-reported error and poor reproducibility; these data were therefore excluded from quantitative analysis. The quantitative conclusions are limited to 20–40 °C.

### 2.6. Statistical Methods

The experimental program followed a completely randomized, single-factor design in which the mixture type constituted the sole controlled factor at four levels: the unmodified (neat) control mixture and three mixtures modified with PCM at 1, 3 and 5% by weight of the bitumen. Three replicate Marshall specimens were prepared and tested at each level (n = 3), yielding a balanced design of twelve observations. The thermal conductivity was evaluated on a separate four-mixture × five-temperature grid in which every cell was represented by one compacted specimen measured four consecutive times through the same probe channel, giving twenty specimens and eighty readings. Because the four readings within a cell are technical replicates of a single specimen rather than independent experimental units, this design contains no between-specimen replication; analyzing the individual readings as independent observations would constitute pseudoreplication and would yield error degrees of freedom that the experiment does not possess. The thermal-conductivity data are therefore reported descriptively only, and no analysis of variance, post hoc test, or *p*-value is attached to them. The design and its analysis follow standard practice for factorial experiments [44]. The experimental unit was the individual Marshall specimen, each produced from an independently prepared batch, so that the three replicates within every group were statistically independent. Each of the six Marshall response variables, namely air voids (Va), voids in the mineral aggregate (VMAs), voids filled with asphalt (VFAs), Marshall stability, flow and the Marshall quotient (MQ), was summarized by its arithmetic mean, standard deviation and coefficient of variation, and a significance level of α = 0.05 was adopted throughout. All statistical analyses were performed using Minitab^®^ statistical software (version 17.1; Minitab, Inc., State College, PA, USA).

Prior to hypothesis testing, the assumptions underlying the parametric analysis of variance were examined for every response variable. The normality of the ANOVA residuals was assessed with the Anderson–Darling test [45], and the homogeneity of variances across the four mixture groups with Levene’s test [46], the latter being robust to mild departures from normality and therefore well suited to small, balanced samples. Levene’s test was non-significant for every response variable (all *p* > 0.50), consistent with homogeneity of variances, and the Anderson–Darling test was non-significant for all six variables, providing no evidence of departure from residual normality. Given the balanced design and the robustness of the one-way ANOVA to mild departures from normality, the parametric procedure was retained throughout, and the limited replicate number (n = 3) is acknowledged as a constraint on the statistical power of the assumption tests; these tests should therefore be regarded as a screen for gross violations rather than as confirmation that the assumptions hold exactly. The effect of mixture type on each response variable was then evaluated by one-way analysis of variance (ANOVA), with mixture type as the fixed factor (three and eight degrees of freedom for the factor and the error term, respectively). The magnitude of the factor effect was quantified by eta-squared (η^2^), which is numerically equal to the coefficient of determination (R^2^) reported by Minitab, and by the less-biased omega-squared (ω^2^) obtained from the analysis-of-variance sums of squares [47]; effect sizes were interpreted against the conventional benchmarks of Cohen [48].

When the ANOVA indicated a statistically significant effect of mixture type (*p* < 0.05), pairwise differences among the group means were resolved using Tukey’s honestly significant difference (HSD) method [49], which controls the family-wise error rate across all comparisons. The multiple-comparison results are reported as Tukey simultaneous 95% confidence intervals for differences between means and, equivalently, through grouping information in which mixture groups that do not share a common letter differ significantly at the 0.05 level (compact-letter notation). Throughout the analysis, a clear distinction is maintained between the experimental results (the measured values and the outcomes of the statistical tests) and their interpretation, and no inference is drawn beyond what the statistical evidence supports.

Because the analysis comprised six separate one-way ANOVAs, one for each Marshall response variable, the multiplicity of tests could, in principle, inflate the family-wise Type I error rate across these omnibus tests. To verify that the conclusions are robust to this issue, the six ANOVA *p*-values were adjusted using the Holm–Bonferroni step-down procedure [50]. All six tests remained statistically significant after adjustment: the two largest Holm-adjusted *p*-values were 0.017 for VMA and 0.006 for flow, and the remaining four were below 0.001. The correction therefore does not alter any conclusions drawn from the individual analyses; this robustness stems directly from the very small unadjusted *p*-values for most response variables. The full analysis-of-variance and Tukey tables are given in the Supplementary Material. It should be noted that the six response variables are not mutually independent: VFA is an algebraic function of Va and VMA (VFA = 100 × (VMA − Va)/VMA), and the Marshall quotient is the ratio of stability to flow. The Holm–Bonferroni adjustment across the six omnibus tests is therefore conservative and is reported solely as a robustness check on the omnibus conclusions rather than as evidence of six independent effects; the primary measured responses are the air voids, VMA, stability and flow, with VFA and the Marshall quotient retained as derived engineering indices. Within each response variable, the Tukey HSD procedure controls the family-wise error rate across pairwise comparisons.

For thermal conductivity, the dependence on temperature and capsule content is summarized descriptively. Each cell is reported as the mean of the four consecutive readings obtained from its single specimen, with the standard deviation of those four readings as a measure of reading-to-reading repeatability under the applied protocol; this standard deviation characterizes the stability of the transient-probe reading on one specimen and is not an estimate of between-specimen variability, nor of the absolute accuracy of the instrument, which the manufacturer states as ±(5% + 0.01 W/m·K). Ordinary least-squares lines fitted to the cell means against temperature and against capsule content are reported solely as compact summaries of the ordered trends; they are not used to test hypotheses and are not interpreted as evidence for a single physical mechanism.

## 3. Results and Discussion

### 3.1. Morphological and Chemical Characterization

**Figure 4:**
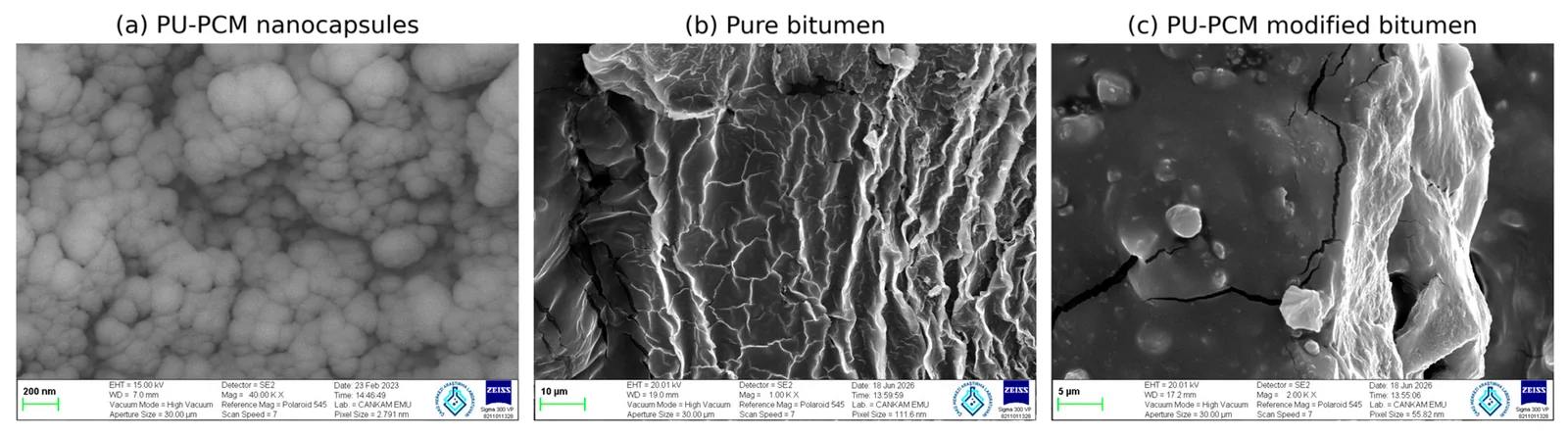
SEM images of (**a**) the PU-PCM nanocapsules, (**b**) the pure bitumen and (**c**) the PU-PCM-modified bitumen (scale bars: 200 nm, 10 µm and 5 µm, respectively).

Figure 4 shows the SEM images of (a) the PUA-PCM nanocapsules, (b) the pure bitumen, and (c) the PUA-PCM-modified bitumen. The particles in panel (a) are approximately spherical and densely packed, with apparent diameters of about 100–300 nm in this micrograph. A quantitative particle-size distribution was not determined from the present SEM image. The nanoscale designation is not based solely on this micrograph: the same synthesis route was characterized previously by the authors using electron microscopy and particle-size analysis, which gave capsule diameters of 55–165 nm with a mean of about 100 nm for the 1500 rpm emulsification condition used here, and 115–365 nm with a mean of about 225 nm for a lower emulsification rate of 1000 rpm [27]. Separate batches prepared by the same route have been reported at 55–175 nm with a mean of about 103 nm and, in a more recent preparation, at a mean of 120 ± 41 nm [25,26,29], so the nanoscale range is reproducible across batches while the exact distribution depends on the emulsification conditions. The apparent diameters observed in the present micrograph are larger than the previously measured range, which is attributed to partial agglomeration of the dried powder and to the difficulty of separating individual capsules from clusters in a single field of view; the present image is therefore used as morphological confirmation of the material applied in this asphalt study rather than as a quantitative size measurement. The pure bitumen exhibits a relatively smooth fracture surface, whereas discrete capsule-like particles are visible in the modified bitumen matrix; these observations support the incorporation of the capsule material but do not, by themselves, establish post-compaction survival of every capsule.

**Figure 5:**
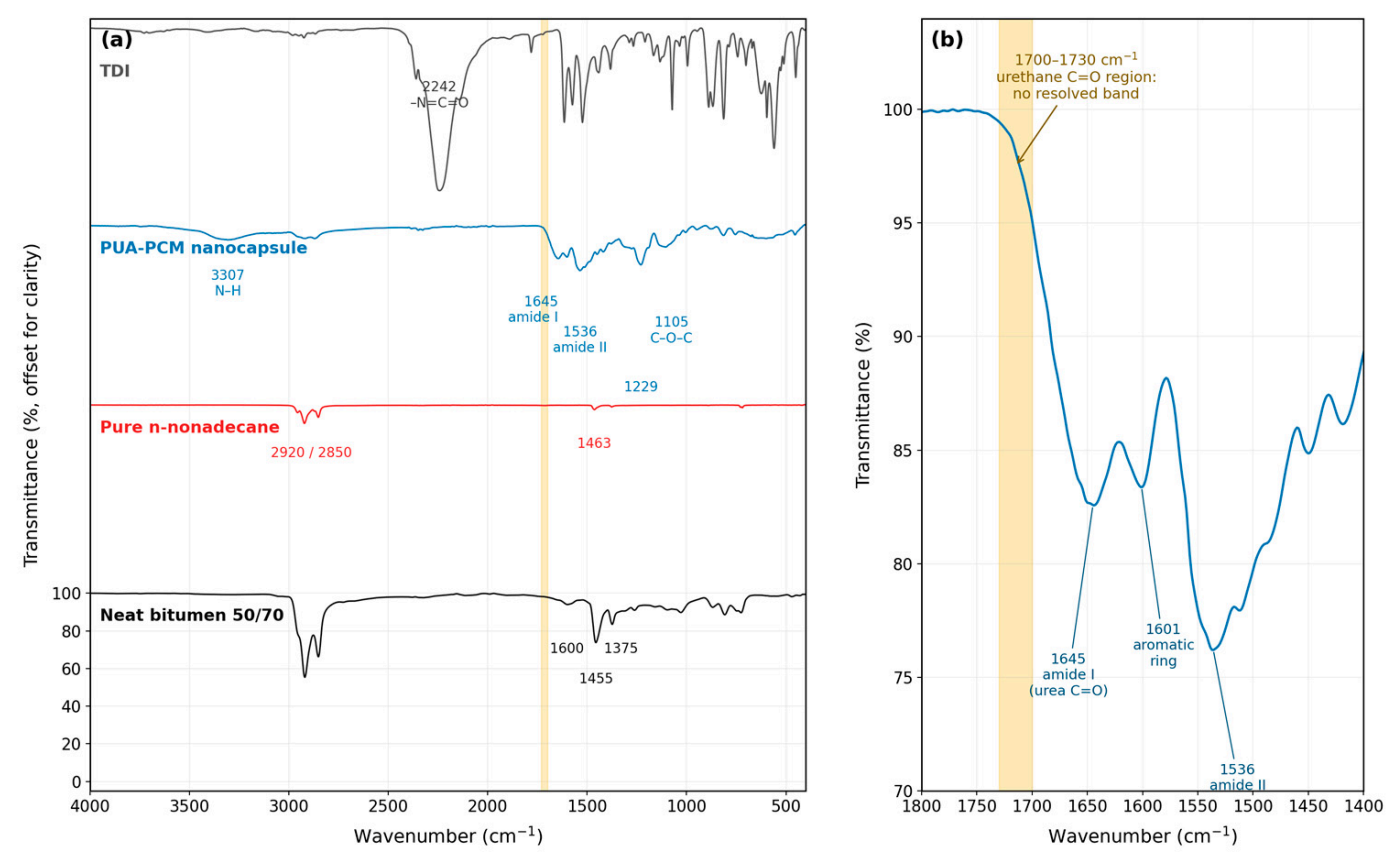
FTIR spectra. (**a**) TDI, the PUA-PCM nanocapsules, pure n-nonadecane and the neat 50/70 bitumen (curves offset for clarity); the strong –N=C=O band of TDI at 2242 cm^−1^ is absent from the capsule spectrum. (**b**) Detail of the capsule spectrum between 1800 and 1400 cm^−1^. The shaded interval in both panels marks the 1700–1730 cm^−1^ urethane carbonyl region, in which no band is resolved.

The FTIR spectra are given in Figure 5. The neat bitumen shows the aliphatic C–H stretching bands at 2920 and 2850 cm^−1^, the aromatic C=C band at 1600 cm^−1^ and the C–H bending bands at 1455 and 1375 cm^−1^, and pure n-nonadecane shows only the C–H stretching bands at 2956, 2922 and 2852 cm^−1^ together with the C–H bending band at 1463 cm^−1^. The capsule spectrum contains a broad N–H stretching band at 3307 cm^−1^, a carbonyl band at 1645 cm^−1^, an aromatic ring band at 1601 cm^−1^, a strong band at 1536 cm^−1^, a band at 1229 cm^−1^, and a C–O–C band at 1105 cm^−1^. Two features identify the shell. First, the carbonyl position is diagnostic: a urea carbonyl (amide I) absorbs at 1630–1660 cm^−1^, whereas a urethane carbonyl appears at 1700–1730 cm^−1^. No band is resolved in the 1700–1730 cm^−1^ interval (Figure 5b); second-derivative analysis of the capsule spectrum returns no component above 1693 cm^−1^, and the residual absorbance in that window falls from 0.020 at 1700 cm^−1^ to 0.002 at 1730 cm^−1^, consistent with the high-frequency wing of the 1645 cm^−1^ band rather than with a separate carbonyl. The 1645 cm^−1^ band, together with the N–H stretch at 3307 cm^−1^ and the band at 1536 cm^−1^, which is assigned to amide II (N–H bending coupled with C–N stretching), is the characteristic amide I/amide II/N–H set of a urea-linked polymer. Second, the isocyanate stretching band of TDI at 2242 cm^−1^, which is the strongest feature of the precursor spectrum, is absent from the capsule spectrum, where the 2200–2320 cm^−1^ region is featureless. The shell-forming reaction therefore consumed the isocyanate groups, and no residual –NCO remains in the washed and dried product. The spectroscopic evidence is thus consistent with the isocyanate–amine reaction route described in Section 2.2 and provides no evidence of a urethane carbonyl. The spectra were not used to demonstrate capsule incorporation into the binder, and the modified-binder spectrum is not shown, because at the dosages used the capsule contribution is not separable from ordinary measurement-to-measurement variation. In the capsule spectrum, the amide I and N–H bands have absorbances of 0.083 and 0.034; at 3% by binder mass, their contributions to a modified-binder spectrum would be approximately 0.0025 and 0.0010 absorbance units, against a neat-bitumen absorbance of 0.016 at the same position, that is an increase of roughly 15% in an already weak band. Two independent attenuated-total-reflectance measurements of the same neat bitumen in this study differ by up to 0.0017 absorbance units over 1500–1750 cm^−1^, which is of the same order. The presence or absence of capsule bands in a 3% modified binder therefore could not be interpreted as evidence either way, and incorporation of the capsule material is assessed instead from the scanning electron micrographs (Figure 4) and from the phase-change response of the modified system (Section 3.2). Accordingly, the FTIR result is reported as identification of the shell polymer, not as a bond-level determination of capsule–binder interaction or post-compaction capsule survival [3,22,25,26,27].

### 3.2. Thermal Analysis (Dsc and Tga)

**Figure 6:**
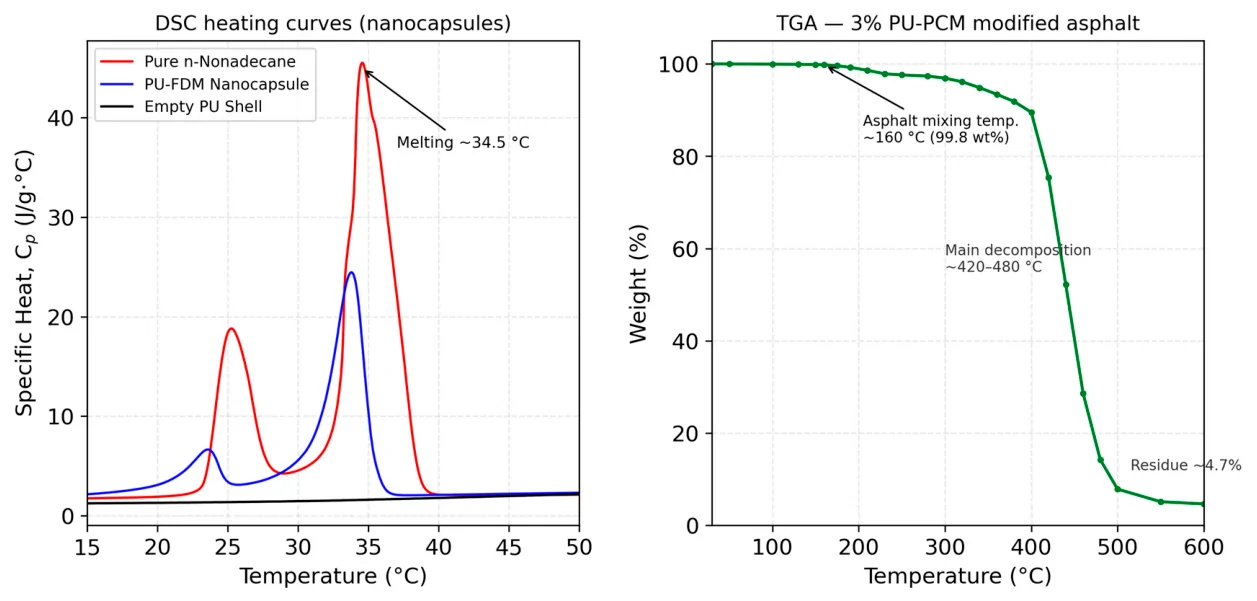
DSC heating curves of the nanocapsules (**left**) and TGA curve of the 3% PU-PCM-modified asphalt (**right**).

Figure 6 shows the DSC heating curves of the PUA-PCM nanocapsule, the pure n-nonadecane core, and the empty-shell reference, along with the TGA curve of the 3% PUA-PCM-modified asphalt. Pure n-nonadecane exhibits two endothermic transitions within the range of interest, a solid–solid (rotator-phase) transition at about 25 °C and melting at 34.5 °C; the corresponding capsule transitions occur at about 23.6 and 33.8 °C. Both transitions fall inside the 20–40 °C interval over which the mixtures were characterized, so the enthalpies are reported as the total endothermic heat integrated over that interval, as given by the instrument: 212.2 J/g for pure n-nonadecane and 92.8 J/g for the capsule. The ratio 92.8/212.2 corresponds to an enthalpy-based encapsulation ratio of approximately 44%. Integrating the melting peak alone gives 148 and 64 J/g and a ratio of approximately 43%, so the value does not depend appreciably on the integration convention provided that the same convention is applied to both materials; the value also agrees with the ratio of about 45% reported previously for this capsule system [26,27]. This term is used here rather than “encapsulation efficiency” because the calculation is enthalpy-based and describes the composition of the capsules rather than the fate of the charged core material. Combining the enthalpy-based ratio with the batch mass balance gives a separate mass-based estimate: the dried product (5.8 g) contains approximately 0.44 × 5.8 ≈ 2.55 g of n-nonadecane, corresponding to about 64% of the 4 g of n-nonadecane charged. The remaining ~3.25 g of product is attributed to the shell, along with any retained surfactant, which is about 81% of the 4 g of TDI and DETA charged. The two quantities are therefore distinct and are reported separately: the enthalpy-based ratio (~44%) describes how much of the capsule mass is phase-change material, whereas the mass-balance value (~64%) describes how much of the charged core was encapsulated and retained after filtration, washing and drying. At the binder/capsule-addition scale, the corresponding nominal enthalpy contributions are approximately 0.93, 2.79 and 4.64 J/g for the 1, 3 and 5% additions, respectively. When expressed on a whole-mixture mass basis using the fixed 6.0% binder content, the approximate latent-heat contributions are much smaller: 0.056, 0.167 and 0.279 J/g mixture for PCM-1, PCM-3 and PCM-5, respectively. These whole-mixture estimates are mass-balance calculations rather than direct DSC measurements of the complete asphalt mixtures and indicate that the direct latent-heat contribution at mixture scale is modest. The empty-shell reference shows no distinct n-nonadecane melting peak. In the TGA curve of the 3% modified asphalt, mass loss up to ~160 °C is less than 0.3%, indicating no appreciable thermal decomposition below the asphalt mixing temperature. TGA probes thermal decomposition only and does not establish mechanical survival of capsules under high-shear mixing or compaction. Verification of post-processing mechanical integrity would require dedicated recovery/microscopy or retained-latent-heat measurements after full asphalt processing.

### 3.3. Thermal Conductivity of the Mixtures

Table 2 shows effective thermal conductivity of the compacted mixtures as a function of temperature and PCM content (W/m·K). Each entry is the mean ± standard deviation of four consecutive readings taken through the same preformed probe channel of a single compacted specimen (one specimen per mixture–temperature cell; twenty specimens in total). The standard deviation describes reading-to-reading repeatability and not between-specimen variability or instrument accuracy. The Supplementary Material provides the complete reading-level records.

**Figure 7:**
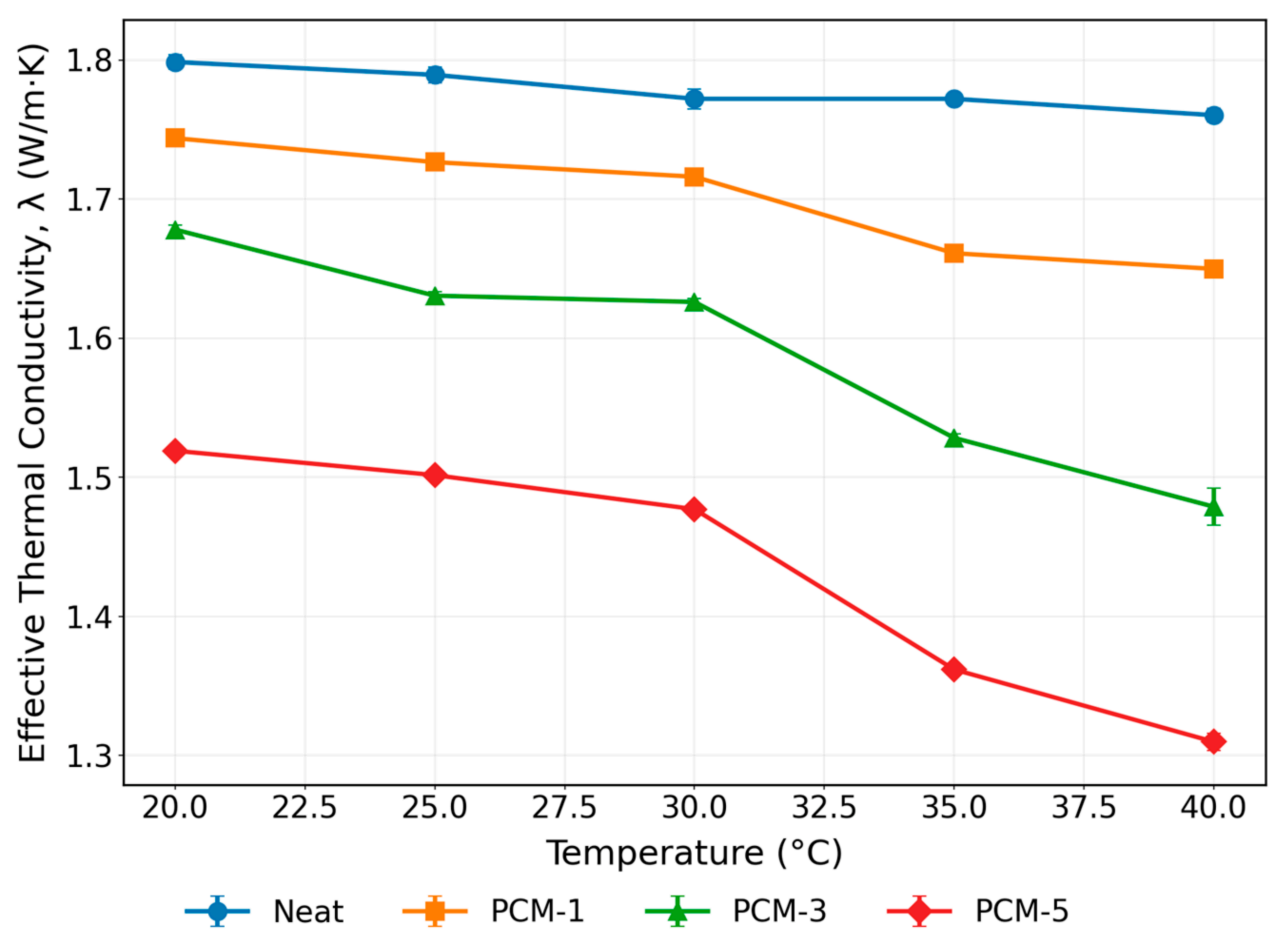
Effective thermal conductivity versus temperature for the four mixture groups. Error bars show ± one standard deviation from four consecutive readings taken on a single specimen in each mixture–temperature cell. Each temperature point corresponds to a separate specimen; connecting lines are a visual guide only and do not represent repeated heating of the same specimen.

The effective thermal conductivity measured under the applied transient-probe protocol decreased with both temperature and capsule content (Table 2, Figure 7). At 40 °C, the neat mixture measured 1.760 W/m·K, while PCM-1, PCM-3 and PCM-5 measured 1.650, 1.479 and 1.310 W/m·K, respectively; the PCM-5 value is approximately 26% lower than the neat-mixture value. The measured values lie within the range of approximately 1.0–2.5 W/m·K reported for dense-graded asphalt mixtures [43,51,52]. Because the total capsule fraction in the asphalt mixture is small and shell-only/free-core asphalt controls were not included, this magnitude is interpreted cautiously and is not assigned to a single mechanism. The observed response may reflect the combined influence of capsule incorporation, constituent thermal properties, phase-transition effects, and changes in the effective microstructure/volumetric state of the compacted mixtures. A reduction in effective thermal conductivity should not be interpreted directly as evidence of pavement cooling or reduced peak surface temperature. Field-scale temperature response depends on conduction, heat capacity, latent-heat storage, boundary conditions, and heating–cooling history, none of which were comprehensively evaluated here. In particular, the capsule melting peak occurs at approximately 33.8 °C, close to the 35 °C measurement level. The transient response in this region may therefore include contributions from both heat conduction and latent-heat absorption; the present experiment was not designed to separate these effects quantitatively. For this reason, the values are reported as effective thermal conductivity under the specified transient-probe protocol rather than as pure intrinsic conductivity constants. Because each mixture–temperature cell was represented by a single specimen, no inferential test was applied to these data, and the trends are described numerically rather than tested. Ordinary least-squares lines fitted to the cell means decreased approximately linearly with capsule content at every temperature (R^2^ = 0.96–0.998), with the slope steepening from about −0.054 W/m·K per % at 20 °C to about −0.089 W/m·K per % at 40 °C, and also decreased approximately linearly with temperature for every mixture (R^2^ = 0.91–0.94), the fitted slope steepening from −0.0019 W/m·K per °C for the neat mixture to −0.0050, −0.0100 and −0.0112 W/m·K per °C for PCM-1, PCM-3 and PCM-5. Relative to the neat mixture, the measured reduction grew from 15.5 to 25.6% for PCM-5 between 20 and 40 °C. These lines summarize the ordered trends and do not represent evidence of a single physical mechanism. The reported standard deviations are calculated from the four consecutive readings of each specimen; they describe reading-to-reading repeatability under this protocol and are substantially smaller than the manufacturer-stated instrument accuracy of ±(5% + 0.01 W/m·K), which therefore governs the absolute uncertainty of the values. The greatest temperature-dependent change in the PCM-containing groups occurred across the temperature interval that includes the capsule phase transition, but this correspondence is treated descriptively rather than as proof of a specific latent-heat mechanism. Independent validation with a second thermal-conductivity method would strengthen mechanistic interpretation.

### 3.4. Marshall Results: Group Means and Post Hoc Groupings

**Figure 8:**
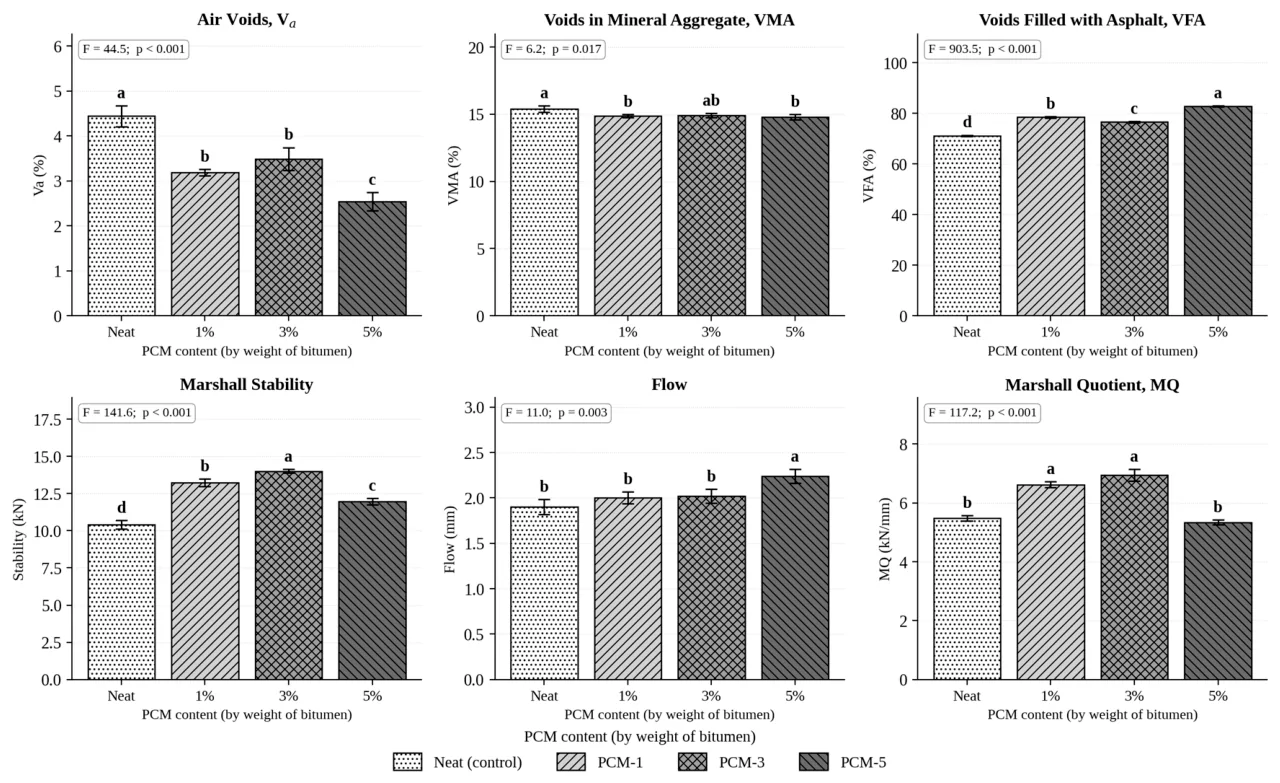
Marshall design parameters as a function of PCM content (mean ± SD, n = 3). Bars sharing a common letter are not significantly different (Tukey HSD, α = 0.05).

Figure 8 summarizes the mean value (±one standard deviation) of each Marshall response variable for the four mixture groups, with the Tukey HSD compact-letter grouping annotated above each bar. The within-group scatter is small, and the mixture groups separate clearly for most parameters. Two features are immediately apparent: PCM addition lowers air voids, reaching a minimum at PCM-5, although the reduction is not strictly monotonic (PCM-1 and PCM-3 do not differ significantly), while Marshall stability and the Marshall quotient respond non-monotonically, peaking at PCM-3. The corresponding numerical values (mean, standard deviation, coefficient of variation, and Tukey grouping) are reported in Table 3.

### 3.5. Significance and Effect Size of the Mixture Factor

**Figure 9:**
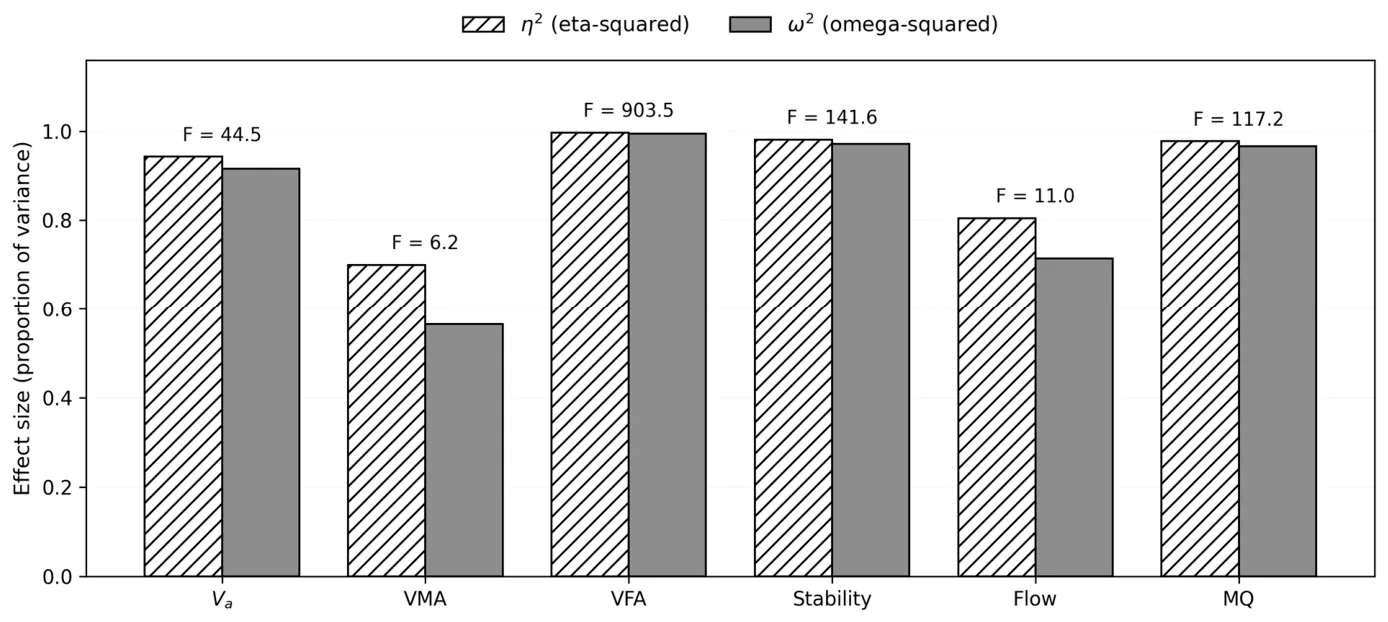
Effect size (η^2^ and ω^2^) of the mixture factor for each Marshall response variable. The F-statistic of the corresponding one-way ANOVA (df = 3, 8) is annotated above each pair. Effect sizes are reported descriptively and are not classified against conventional magnitude thresholds.

**Figure 10:**
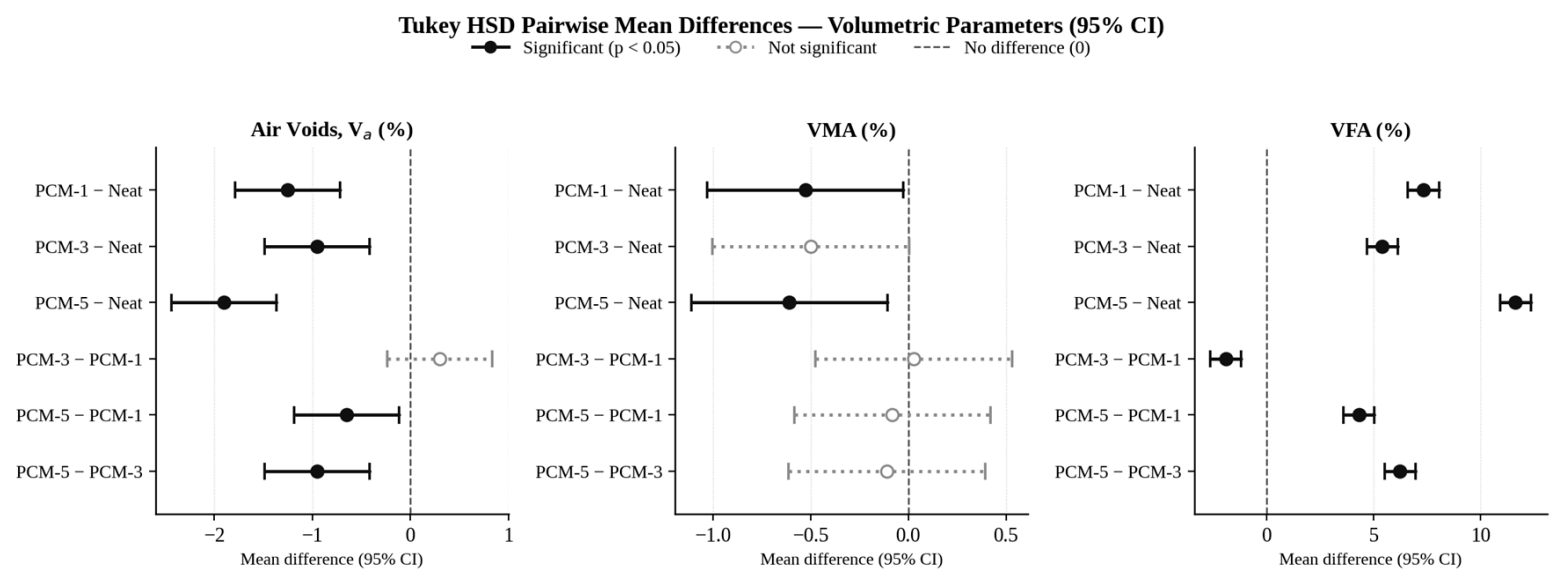
Tukey HSD pairwise mean differences (95% confidence intervals) for the volumetric parameters (air voids, VMA, VFA). A solid line with a filled marker denotes a statistically significant difference (the interval does not cross zero); a dotted line with an open marker denotes a non-significant difference. Each difference is computed as the first-named minus the second-named mixture, so a positive value indicates the higher mean for the first-named mixture.

**Figure 11:**
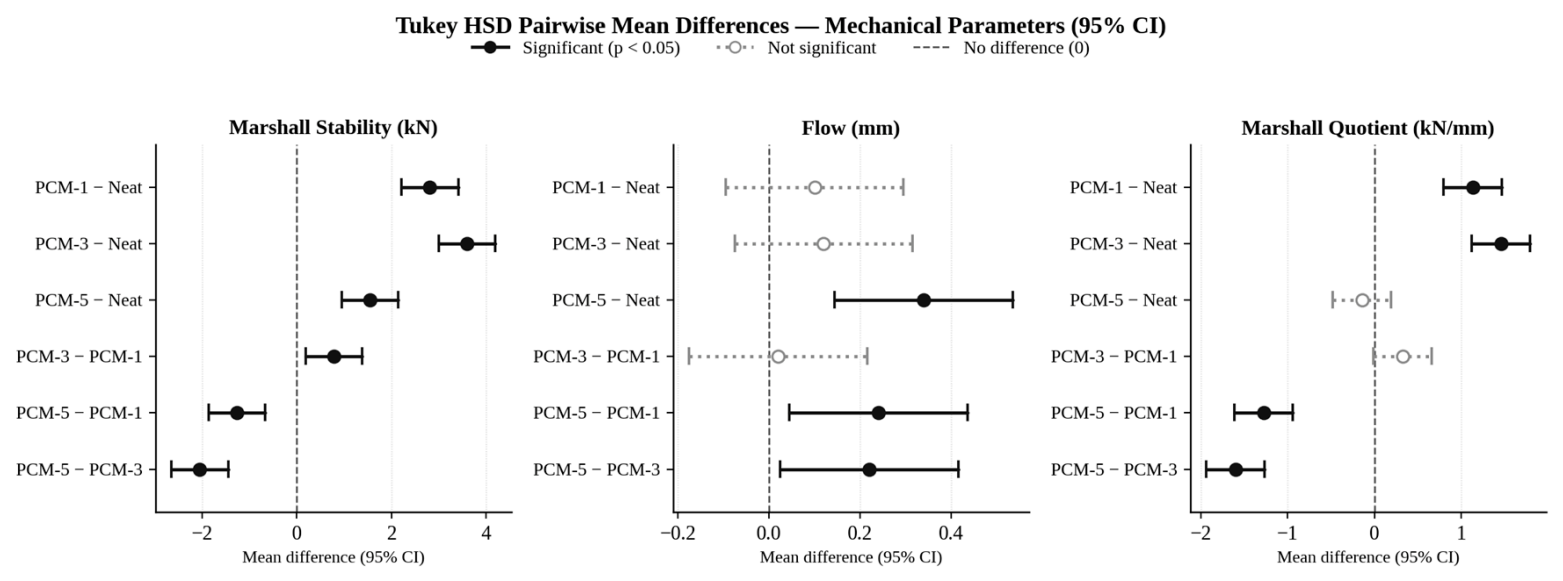
Tukey HSD pairwise mean differences (95% confidence intervals) for the mechanical parameters (Marshall stability, flow, Marshall quotient). A solid line with a filled marker denotes a statistically significant difference (the interval does not cross zero); a dotted line with an open marker denotes a non-significant difference. Each difference is computed as the first-named minus the second-named mixture, so a positive value indicates the higher mean for the first-named mixture.

The one-way ANOVA showed that the mixture factor had a statistically significant effect on every response variable (*p* < 0.05 in all cases; df = 3, 8). The corresponding F-statistics and *p*-values were F = 44.5 (*p* < 0.001) for air voids, 6.2 (*p* = 0.017) for VMA, 903.5 (*p* < 0.001) for VFA, 141.6 (*p* < 0.001) for stability, 11.0 (*p* = 0.003) for flow and 117.2 (*p* < 0.001) for the Marshall quotient. The effect-size estimates in Figure 9 summarize the observed between-group variation relative to within-group variation in this dataset. They do not establish practical superiority or isolate an intrinsic PCM effect from accompanying volumetric and workability differences. Interpretation therefore focuses on the magnitudes and Tukey simultaneous 95% confidence intervals of the pairwise differences presented in Figure 10 and Figure 11. Significant results across the six responses should not be regarded as six independent confirmations, because VFA and MQ are derived indicators.

### 3.6. Pairwise Comparisons (Tukey HSD)

Figure 10 and Figure 11 show Tukey HSD pairwise mean differences with 95% confidence intervals, separated into volumetric and mechanical parameters, respectively. A comparison is statistically significant when its confidence interval does not cross the zero line (solid line with a filled marker); non-significant comparisons are shown as dotted lines with open markers. For the volumetric parameters (Figure 10), all PCM mixtures differ significantly from the neat control in air voids and VFA; for air voids, the three PCM dosages are mutually distinguishable except for the PCM-1/PCM-3 pair, and for VFA, every group differs significantly from every other, whereas for VMA, only PCM-1 and PCM-5 are significantly lower than the control (PCM-3 is not). The three PCM dosages do not differ among themselves. For the mechanical parameters (Figure 11), Marshall stability differs significantly between every dosage pair, with PCM-3 the highest; the Marshall quotient is highest at PCM-1 and PCM-3, which are statistically indistinguishable from each other, whereas PCM-5 is indistinguishable from the neat control; and flow is the least sensitive parameter, with only the PCM-5 mixture differing significantly from the control and from the two lower dosages.

### 3.7. Volumetric Parameters (Va, Vma, Vfa)

*Results:* PCM addition significantly reduced the air-void content relative to the control, from 4.44% (Neat) to a minimum of 2.54% at PCM-5, which differed significantly from all other groups (Figure 10). VMA was significantly lower than the control for PCM-1 and PCM-5, whereas PCM-3 did not differ significantly from the control; the three PCM dosages did not differ among themselves. VFA increased markedly with PCM modification, and every group differed significantly from every other, with PCM-5 exhibiting the highest value (82.84%).

*Interpretation:* under the common 6.0% binder-content and 150 °C preparation condition, the capsule-containing mixtures exhibited lower air-void contents, generally lower VMA, and higher VFA than the neat control. These differences describe the measured volumetric response but should not be interpreted as an intrinsic densification mechanism attributable solely to the capsules. Because the binder content was held constant at the 6.0% reference value established for the neat mixture, part of the volumetric shift is attributable to the additional binder-phase volume introduced by capsule addition, and the present design cannot isolate an intrinsic effect independent of binder content. In addition, because all formulations were prepared at the same nominal temperature without independent viscosity-based workability matching, differences in binder rheology and compactability may have contributed to the observed volumetric response. Assessed against the stated Marshall design criteria, the modified mixtures should therefore not be described as unqualified improvements, and binder content should be re-optimized at each capsule dosage before assessing design compliance.

### 3.8. Mechanical Parameters (Stability, Flow, Mq)

*Results:* Marshall stability responded non-monotonically to dosage (Figure 8 and Figure 11): all PCM mixtures showed significantly higher measured Marshall stability than the control, and stability reached its maximum at PCM-3 (14.01 kN), significantly higher than both PCM-1 and PCM-5, before declining at 5%. Flow increased only modestly overall; PCM-5 showed the highest value (2.24 mm) and was the only group significantly different from the control. The Marshall quotient was highest at PCM-3 (6.94 kN/mm) and PCM-1 (6.62 kN/mm), which were statistically indistinguishable from each other, whereas the value at PCM-5 (5.34 kN/mm) was statistically indistinguishable from the neat control.

*Interpretation:* under the fixed 6.0% binder-content and common compaction condition, PCM-3 exhibited the highest measured Marshall stability and a Marshall quotient among the highest values, while flow did not differ significantly from the control at the lower dosages. The differing stability and flow responses are not contradictory: the mixtures sustained higher maximum loads without a statistically detectable increase in the associated deformation at PCM-1 and PCM-3. Mean flow increased slightly from 1.90 mm in the control to 2.00 and 2.02 mm, respectively, but these differences were not significant in the Tukey comparisons. This finding indicates a greater change in peak load than in flow under the applied test conditions, rather than no effect on deformation. These results should not be interpreted as proof of intrinsic material strengthening or comprehensive rutting/deformation resistance because the mixtures also differed in air-void content and other volumetric properties. The measured stability response may reflect a combined influence of capsule incorporation, effective mastic volume, workability/compactability, and mixture density. Applying the same compaction effort did not produce matched air-void contents, so the contribution of volumetric differences cannot be separated from that of capsule addition. Although PCM-5 had the lowest air-void content, its stability was lower than that of PCM-3; thus, air-void reduction alone does not sufficiently explain the dosage-dependent response. Matched-density comparisons, binder rheology, or additional performance tests would be required to isolate an intrinsic strengthening mechanism. At PCM-5, stability and MQ decreased relative to PCM-3 and flow increased, indicating that the measured Marshall response was not monotonic with capsule dosage.

### 3.9. Comparison of the Tested PCM Contents

The thermal-conductivity and Marshall responses did not vary in the same direction across all capsule dosages. At 40 °C, the measured effective thermal conductivity was approximately 6, 16, and 26% lower than the neat mixture for PCM-1, PCM-3, and PCM-5, respectively, whereas Marshall stability reached its highest measured value at PCM-3 and decreased at PCM-5. The volumetric results also shifted with capsule content, and the modified mixtures were not independently optimized at each dosage. Accordingly, no dosage is identified here as an “optimum” or fully design-compliant formulation. Under the fixed 6.0% bitumen-content condition, PCM-3 exhibited the highest measured Marshall stability and lower measured effective thermal conductivity than the neat mixture. However, its VFA exceeded the adopted upper limit, and none of the PCM-modified formulations satisfied all the adopted volumetric criteria. These results therefore do not establish an optimum PCM dosage or a design-compliant modified mixture. Re-determining the reference binder content for each capsule dosage is required before any formulation can be considered a finalized mix design [31].

### 3.10. Comparison with the Literature

Table 4 compares the present nanocapsules and their effects with representative studies on PCM-modified bitumen and asphalt. The core melting temperature (34.5 °C; 33.8 °C for the capsule) and the n-nonadecane core are consistent with Sarı et al. [22], who reported 31.2 °C and a latent heat of 139.2 J/g for a PMMA/n-nonadecane capsule; the lower capsule enthalpy obtained here (92.8 J/g) reflects a heavier polyurea shell and a correspondingly lower core mass fraction. The thermal stability up to ~160 °C agrees with Betancourt-Jimenez et al. [20], who found that microcapsules largely survived asphalt mixing at 140 °C and produced a 5–10 °C temperature lag in hot-mix asphalt. The higher measured Marshall stability at the lower/intermediate capsule contents can be compared qualitatively with binder-scale reports of altered stiffness or deformation-related response [17], while the decrease in measured effective thermal conductivity follows the direction reported in other PCM-modified asphalt studies [12,30]. These comparisons are qualitative because the materials, test methods, and scales differ. The distinctive contributions of the present work are the use of nanoscale (rather than microscale) capsules and the joint evaluation of effective thermal conductivity and Marshall response for the same mixture formulations using separate compacted specimen sets.

### 3.11. Limitations

The PCM mixtures were compared with the control at the fixed 6.0% reference bitumen content established for the neat mixture, so the volumetric response reflects both capsule addition and changes in effective binder/mastic volume; this design cannot isolate an intrinsic capsule effect independent of binder content. The replicate number for the Marshall program was three per group, which limits the power of assumption checks. The Marshall test characterizes mixtures at a single temperature (60 °C) and cannot, by itself, establish comprehensive pavement performance, rutting resistance, fatigue resistance, or service-life improvement. The mixtures were prepared at a common nominal temperature without viscosity-based workability matching, so rheological/compactability differences may contribute to the observed volumetric response. The surface-temperature buffering capacity was not measured directly. Effective thermal conductivity was quantified only over 20–40 °C using a transient needle-probe method; measurements above 40 °C became unstable as the asphalt softened and were not retained for quantitative analysis. Measurements close to the ~33.8 °C capsule melting interval may include a latent-heat contribution to the transient thermal response, and no independent steady-state or second-method validation was performed. Each mixture–temperature cell of the thermal-conductivity program was represented by a single compacted specimen measured four consecutive times through one probe channel, so the program contains technical repetition but no between-specimen replication. The mixture and temperature effects on effective conductivity therefore cannot be separated statistically from specimen-to-specimen manufacturing variability, and no inferential analysis of these data is reported; the reported differences are descriptive and would require replicated specimens for formal testing. A dedicated heating–cooling hysteresis protocol was likewise not performed. The whole-mixture latent-heat contribution calculated from the capsule enthalpy and dosage is modest. The present study also did not include empty-shell or free n-nonadecane asphalt controls, so the individual contributions of shell, core, and particulate addition cannot be separated mechanistically. TGA establishes thermal stability against decomposition but not mechanical survival of capsules after high-shear mixing and compaction. A quantitative particle-size distribution was not determined for the present batch from the current SEM image, although the same synthesis route had been characterized at the nanoscale in earlier work [25,26,27]. Durability under repeated thermal cycling and binder aging was not evaluated. These limitations define the scope of the conclusions and the priorities for future work. Direct binder rheology (e.g., DSR/BBR) was not measured, so no specific rheological mechanism is inferred from the nanoscale capsule size. A simple two-phase effective-medium conductivity model was not applied because the asphalt mixture is a multiphase aggregate–binder–air-void–capsule system and the constituent thermal conductivities, phase continuity, and effective volume fractions required for a defensible model were not independently determined. A formal techno-economic or life-cycle cost analysis was also outside the scope of this study; scale-up would require evaluating synthesis yield, reagent/solvent demand, washing and drying steps, process simplification, and production cost before field implementation.

## 4. Conclusions

Polyurea-shelled, n-nonadecane-core PCM nanocapsules prepared using a previously developed interfacial polymerization route were incorporated into bitumen at 1, 3, and 5% by weight of the binder and evaluated at capsule/binder and mixture scales. The principal findings are as follows:

- SEM showed approximately spherical capsule particles in the present material, while the nanoscale designation is additionally supported by earlier particle-size and electron-microscopy characterization of the same synthesis route [25,26,27,29]. FTIR identified the shell as a urea-linked polymer through the amide I, amide II, and N–H bands, the absence of a resolved urethane carbonyl, and the complete consumption of the isocyanate band of the precursor; it was not used to quantify capsule content in the binder or to establish post-compaction capsule survival.
- DSC showed melting at 34.5 °C for pure n-nonadecane and at ~33.8 °C for the capsule, with a total capsule endothermic enthalpy of 92.8 J/g over the 20–40 °C interval and an enthalpy-based encapsulation ratio of ~44%. The corresponding nominal whole-mixture latent-heat contributions were ~0.056, 0.167, and 0.279 J/g mixture for PCM-1, PCM-3, and PCM-5, respectively. TGA showed no appreciable thermal decomposition at the 150 °C preparation temperature but does not establish mechanical capsule survival during mixing and compaction.
- The effective thermal conductivity measured by the KD2 Pro/KS-1 transient-probe method decreased with temperature and capsule content within the tested 20–40 °C range, reaching 1.31 W/m·K for PCM-5 versus 1.76 W/m·K for the neat mixture at 40 °C. This result is reported as an effective transient-probe response; field-scale pavement cooling and pure intrinsic conductivity were not directly established.
- One-way ANOVA showed statistically significant differences among the tested mixture groups for the reported Marshall response variables (*p* < 0.05), with large effect-size estimates; however, the Marshall program used n = 3 per group, and VFA and MQ are derived indices rather than independent primary measurements.
- Under the common 6.0% binder-content condition, the PCM-containing mixtures exhibited lower air-void contents and higher measured Marshall stability than the neat control, with the maximum stability observed at PCM-3. These changes occurred alongside shifts in volumetric properties and therefore should not be interpreted as evidence of intrinsic reinforcement independent of density, workability, or binder-content effects.
- None of the PCM-modified mixtures satisfied all the adopted volumetric design criteria at the fixed 6.0% bitumen content. Although PCM-3 exhibited the highest measured Marshall stability, its VFA exceeded the specified upper limit. Dosage-specific binder-content optimization and verification of all design criteria are required before recommending a PCM-modified formulation.

Overall, the present study shows measurable changes in effective thermal conductivity and Marshall response after incorporating the previously developed n-nonadecane capsule system. The results do not directly demonstrate pavement cooling, field-scale thermal regulation, comprehensive rutting resistance, or preserved overall pavement performance. Future work should re-determine reference binder content at each dosage, measure temperature buffering directly at mixture/pavement scale, evaluate rheology and compactability, verify post-processing capsule integrity, and assess durability under thermal cycling and aging. Future work should also include mechanistic thermal-conductivity modeling where sufficient constituent-property data are available, direct binder rheology, and techno-economic/scale-up assessment of capsule production.

## Figures and Tables

**Table 1:** Physical properties of the bitumen and aggregate.

Property	Standard	Value
Penetration	ASTM D5 [32]	52
Specific gravity	ASTM D70 [33]	1.032
Bulk specific gravity (total aggregate)	ASTM C127/C128 [34,35]	2.663
Apparent specific gravity (total aggregate)	ASTM C127/C128 [34,35]	2.803
Effective specific gravity (total aggregate)	ASTM D2041 [36]	2.709
Asphalt absorption	Calculated	0.66
Coarse aggregate, bulk specific gravity	ASTM C127 [34]	2.684
Coarse aggregate, apparent specific gravity	ASTM C127 [34]	2.819
Fine aggregate, bulk specific gravity	ASTM C128 [35]	2.639
Fine aggregate, apparent specific gravity	ASTM C128 [35]	2.812
Mineral filler, apparent specific gravity	ASTM D854 [37]	2.646

**Table 2:** Thermal conductivity of the compacted mixtures as a function of temperature and PCM content (W/m·K, mean ± standard deviation, n = 5).

Temperature (°C)	Neat	PCM-1	PCM-3	PCM-5
20	1.798 ± 0.005	1.744 ± 0.003	1.678 ± 0.003	1.519 ± 0.001
25	1.789 ± 0.006	1.727 ± 0.003	1.631 ± 0.003	1.502 ± 0.001
30	1.772 ± 0.007	1.716 ± 0.004	1.626 ± 0.002	1.477 ± 0.003
35	1.772 ± 0.002	1.661 ± 0.002	1.528 ± 0.003	1.362 ± 0.002
40	1.760 ± 0.005	1.650 ± 0.003	1.479 ± 0.013	1.310 ± 0.006

**Table 3:** Descriptive statistics and Tukey groupings for each Marshall response variable (n = 3 per group).

Mixture	Mean ± SD	CV (%)	Group
Air voids, Va (%)			
Neat	4.44 ± 0.236	5.31	a
PCM-1	3.19 ± 0.072	2.26	b
PCM-3	3.49 ± 0.255	7.32	b
PCM-5	2.54 ± 0.207	8.14	c
Voids in mineral aggregate, VMA (%)			
Neat	15.40 ± 0.243	1.58	a
PCM-1	14.87 ± 0.129	0.87	b
PCM-3	14.90 ± 0.161	1.08	ab
PCM-5	14.79 ± 0.215	1.45	b
Voids filled with asphalt, VFA (%)			
Neat	71.20 ± 0.248	0.35	d
PCM-1	78.53 ± 0.291	0.37	b
PCM-3	76.61 ± 0.296	0.39	c
PCM-5	82.84 ± 0.275	0.33	a
Marshall stability (kN)			
Neat	10.42 ± 0.295	2.83	d
PCM-1	13.23 ± 0.245	1.85	b
PCM-3	14.01 ± 0.125	0.89	a
PCM-5	11.96 ± 0.215	1.80	c
Flow (mm)			
Neat	1.90 ± 0.082	4.31	b
PCM-1	2.00 ± 0.066	3.28	b
PCM-3	2.02 ± 0.075	3.74	b
PCM-5	2.24 ± 0.075	3.37	a
Marshall quotient, MQ (kN/mm)			
Neat	5.49 ± 0.090	1.64	b
PCM-1	6.62 ± 0.100	1.51	a
PCM-3	6.94 ± 0.202	2.90	a
PCM-5	5.34 ± 0.084	1.57	b

Values are mean ± standard deviation; CV = coefficient of variation. Within each response variable, means not sharing a common letter in the “Group” column are significantly different (Tukey HSD, α = 0.05).

**Table 4:** Comparison of the present study with representative PCM-modified bitumen/asphalt studies.

Study	Shell/Core	Tm (°C)	Latent Heat (J/g)	Capsule-Related Metric (Not Directly Comparable)	Main Reported Effect
This study	PU/n-nonadecane	34.5 (core)/33.8 (capsule)	62.4 (capsule)	Enthalpy-based ratio ~44%	Mixture λ −26% at 5%; Marshall stability +34% at 3%
Sarı et al. [22]	PMMA/n-nonadecane	31.2	139.2	–	TES characterization
Betancourt-Jimenez et al. [20]	microPCM in HMA	–	~200	Reported survival ~90%	HMA temperature lag 5–10 °C
Wei et al. [17]	microPCM/binder	–	–	–	RTFO/PAV aging; binder performance improved

## Data Availability

The group statistics of the Marshall program are reported in Table 3. The complete reading-level records of the thermal-conductivity measurements, comprising the four consecutive readings and the corresponding recorded specimen temperatures for each of the twenty specimens, are provided as Supplementary Material S1. Supplementary Material S2 provides the differential scanning calorimetry records of the capsule, the pure n-nonadecane core, and the empty-shell reference, including the heating and cooling specific-heat curves; Supplementary Material S3 provides the reading-level infrared spectra of the four materials measured by FTIR.

## Supplementary Materials

The following supporting information can be downloaded at: https://www.mdpi.com/article/10.3390/polym18212635/s1.

## Nomenclature

ANOVA	analysis of variance
CV	coefficient of variation
DETA	diethylenetriamine
DSC	differential scanning calorimetry
FTIR	Fourier-transform infrared spectroscopy
HSD	honestly significant difference
MQ	Marshall quotient
PCM	phase-change material
PU	polyurethane
SEM	scanning electron microscopy
TDI	toluene diisocyanate
TGA	thermogravimetric analysis
Va	air voids
VFA	voids filled with asphalt
VMA	voids in the mineral aggregate
ΔHm	melting enthalpy
η^2^	eta-squared (effect size)
λ	thermal conductivity
ω^2^	omega-squared (effect size)
